# Ultrasound‐assisted synthesis of nanoemulsion/protein blend for packaging application

**DOI:** 10.1002/fsn3.2776

**Published:** 2022-03-11

**Authors:** Fidele Benimana, Irina Y. Potoroko, Prateek Pathak, Shirish H. Sonawane, Shriram Sonawane, Uday D. Bagale

**Affiliations:** ^1^ Department of Food and Biotechnology South Ural State University Chelyabinsk Russia; ^2^ Laboratory of Computational Modeling of Drugs Higher Medical and Biological School South Ural State University Chelyabinsk Russia; ^3^ Department of Chemical Engineering National Institute of Technology Warangal India; ^4^ Department of Chemical Engineering Visvesvaraya National Institute of Technology Nagpur India

**Keywords:** active film, mechanical properties, nanocomposite, sunflower oil, water vapor permeability

## Abstract

In the present work, we studied the formation of sunflower oil nanoemulsion using ultrasound techniques. Later, we investigated the development of active films based on a mixture of whey protein containing sunflower oil base nanoemulsion with different concentrations (10, 25, and 50% of total whey protein). The prepared film was by analyzing using the Fourier transform infrared (FTIR), X‐ray diffraction (XRD), and field‐emission scanning electron microscope (FE‐SEM). The film shows no changes in its integrity and crystallinity compared to the virgin film. The presence of nanoemulsion improves the mechanical properties from 2.75 MPa to 3.52 MPa while it decreases the water vapor permeability from 3.4 × 10^–10^ to 1.3 × 10^−10^g/m.s.Pa for concentrations NE (50% of Whey protein). The antioxidant activity for Tween 20 nanoemulsion is 38.7% compared to 36.1% for Tween 80 nanoemulsion. The antimicrobial activity of the film contains sunflower nanoemulsion higher than virgin films. The results showed the potential of blend film of whey protein with nanoemulsion for active films for novel food protection.

## Graphical abstract

Current research is applicable to protect the food base on packaging material preparation and its characterization in terms of chemical and mechanical properties.

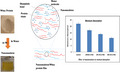

## 1 INTRODUCTION

Since the last decades, packaging film is significant and essential for food products. It helps to enhance and protect the products against environmental conditions during processing and storage(Agudelo‐Cuartas et al., 2020; Dammak et al., 2021; Ncube et al., 2020; RameshKumar et al., 2020; Robertson, 2020). In the early days, petroleum‐based materials are used as packaging material for food products because of their desirable properties, such as strength, flexibility, resistivity. However, the biggest problem with these materials is their degradation and environmental problems. To minimize the use of these products, the implementation of biodegradable materials has gaining interest (Blank et al., 2020; Nagalakshmaiah et al., 2017; Othman, 2014; RameshKumar et al., 2020).

All biodegradable materials, especially protein and its derivatives, provide film‐forming property (RameshKumar et al., 2020; Shendurse et al., 2018). Many hydroxyl groups are connected in protein film, forming hydrogen bonds, and making them strong protein films. The advantage of using whey protein in the packaging field as it has a better barrier, mechanical properties (Calva‐Estrada et al., 2019; Chen et al., 2019; Flôres et al., 2017). To get barrier and antimicrobial properties, many researchers have proposed adding essential oil extracted from plants in the form of nanoemulsion to the packaging application (Otoni et al., 2014; Prakash et al., 2018; Salvia‐Trujillo et al., 2015). Sunflower oil has a health impact benefit, and it is a popular oil in the world. Russian is among the top five makers of sunflower oil production. A high phenolic structure makes him a strong candidate for good antioxidants that can help minimize health‐related diseases. Other research has reported sunflower oil to be antimicrobial (Liu et al., 2020; da Silva et al., 2021). However, a problem with sunflower oil is the poor solubility in water because of its hydrophobic nature. For improving solubility and bioavailability of sunflower oil, encapsulation is the best technique (Akbas et al., 2018; Alexandre et al., 2016; Ghadetaj et al., 2018; Silva et al., 2012; Topuz et al., 2016). The use of the ultrasound approach for food nanoemulsion has gained attention because of its low operation cost, required low energy, ease to handle, and better control over its formulation variables (Akbas et al., 2018; Bagale et al., 2018; Ghosh et al., 2013; Jadhav et al., 2015; Kumar et al., 2017).

There are few articles where biopolymer material and the encapsulated essential oil in the form of nanoemulsion are used as active film packaging (Alexandre et al., 2016; Bahram et al., 2014; Gaikwad et al., 2016; Ghadetaj et al., 2018; Mousavian et al., 2021; Topuz et al., 2016). Sodium alginate, chitosan, starch, and gelatin fish are biopolymer used (Alexandre et al., 2016; Robledo et al., 2018; Topuz et al., 2016; Wu et al., 2015). Alexandre et al., (2016), reported that essential oil‐based nanoemulsion provides antimicrobial as well as antioxidant properties.

To the best of our knowledge, not much research has reported using nanoemulsion and biopolymer. We aim to implement whey protein as a carrier and add it to the sunflower nanoemulsion to form edible film for packaging application. During this work, we mainly focus on preparing sunflower nanoemulsions and their incorporation into a whey protein coating. We have studied physical and chemical properties such as water vapor permeability, moisture, and release of oil. In addition, we studied the antimicrobial and antioxidant tests along with mechanical properties for control and nanoemulsion film.

## 2 MATERIALS AND METHODS

### 2.1 Materials

Whey protein isolate was purchased from Do 4a shop, Chelyabinsk, Russia. Sunflower oil used in this research was purchased from the local supermarket (producer ASTON JSC, Rostov‐on‐Don Russia). Glycerol, Tween 20, Tween80 from the chemical shop of Thermal fisher scientific, Russia, and all the other reagents were used of maker Merk found in laboratory department of food and biotechnology, South Ural State University Chelyabinsk.

### 2.2 Nanoemulsion synthesis and its characterization

As we are formulated, coarse emulsions are based on two different Tween 80 and 20 for stabilization. Tween 20 & 80 were used due to higher HLB values to stabilize emulsion droplets by stearic effect (shielding effect). To preparation of the nanoemulsion method described by Ghadetaj, A et al., 2018 was accomplished. The emulsion was formed by mingling sunflower oil & Tween 80 in a 4:2 ratio. Initially, we have prepared an aqueous solution of Tween 80 and 20 in distilled water, respectively. In this aqueous solution, we add sunflower oil and mix for 20 min at 3 *g* using a magnetic stealer under ambient temperature. After that, the coarse emulsion was treated with ultrasound (Volna UZTA 063/22 OM, Biysk) and a power output of 450 W of 80% amplitude. We kept the solution under sonication for 10 min in a cooling beaker to overcome excess heat during the reaction. After getting the nanoemulsion, we check the particle size distribution and optical microscope image as a confirmation test.

### 2.3 Film preparation

In preparing active biopolymer film, we initially have prepared a solution by dissolving 3 g of whey protein in distilled water as described by (Ghadetaj et al., 2018). Later, we added slight polyvinyl alcohol that acts as colloidal material in the above solution. The above solution was sonicated for 15 min for a homogeneous solution by maintaining the solution's pH at 8 using hydroxide solution. Then, it was stirred for 40 min at 90°C in instruction to denature proteins. Before the addition of glycerol base on protein, the concentration solution allowed was cooling. (Whey protein 2: Glycerol 1). Sunflower oil nanoemulsions were incorporated at different levels of 10, 25, and 50% of whey protein into the film solution. The solutions were dispensed on a glasses plate for drying (24 h) and further analysis.

### 2.4 Analysis of the active film

#### 2.4.1 Thickness

The prepared film has then checked the thickness using a digital micrometer at different positions to analyze the mechanical and water vapor permeability test.

#### 2.4.2 Water Vapor Permeability (WVP)

For food packaging applications, the most critical test is water vapor permeability. It decides its shelf life of food. The shelf life of consumer packages of frozen foods, baked goods, instant coffee, dehydrated foods, fresh produce is dependent directly on the moisture permeability rate of the packaging material used. Glasses of 2 cm in diameter and 4 cm height were used to measure the water vapor permeability of our formed films. Disc shapes of films with a diameter slightly larger than glasses were put on top of glasses containing 3 g of CaSO_4_ to get Relative Humidity of zero. Relative Humidity of 97%, all glasses were placed in a desiccator containing saturated K_2_SO_4_ solution at 25℃, then the weight of the glasses was measured every 24 hr. After that, the weight gained by the film concerning time was recorded and plotted on the graph as described by (Meira et al., 2017).

The below formula obtained WVP:

$$\text{WVP}=\frac{\mathrm{WVTR}}{P\left(R1‐R2\right)}X$$

Where X is the film thickness (m), *P* is the water vapor pressure saturation (Pa) at standard temperature (25°C), R_1_ the RH in a desiccator, R_2_ is RH in the glass. According to these conditions, [P (R_1_−R_2_)] is 3073.93 Pa.

#### 2.4.3 Swelling ratio of films

As described by (Ghadetaj et al., 2018), the Swelling ratio was used to determine how films could absorb water. Film samples of 2 × 2 cm^2^ were submerging in distilled water for 24 h. Once 24 h completed, the obtained wet film samples were tapped with filter paper and check the weight. The swelling ratio was obtained by the following equation 1:

$$\text{Swelling ratio}\;\left(\%\right)=\frac{W2‐W1}{W1}*100\left(1\right)$$

Where *W*_1_ and *W*_2_ are the initial and the final wt of the film.

#### 2.4.4 Moisture absorption of the film

In the modified method described by (Meira et al., 2017), the moisture absorption of film samples was determined, the dried sample films of 2 cm^2^ were conditioned at 0% RH (CaSO_4_) for 24 h., and then placed in desiccators containing CaNO_3_ saturated solution at room temperature to maintain humidity between 50% and 55% RH. The moisture absorption was determined as the percentage of weight reduction of the initial film till equilibrium during drying and reported on a wet basis. The moisture absorbance of films was obtained by equation 2:

$$\text{Moisture absorption\textbackslash{}\%}\;=\frac{Wt‐wo}{\mathrm{wo}}*100\left(2\right)$$

#### 2.4.5 Fourier transform infrared spectroscopy (FTIR)

The FTIR spectrometry (FTIR Q410 Alpha, St. Petersburg) was used to determine the connections of whey protein and sunflower nonemulsion in the films. FTIR of the film sample was to identify the functional group present in the range 4000–400 cm^−1^ wavelength.

#### 2.4.6 Field‐emission scanning electron microscopy (FE‐SEM)

Field‐emission scanning electron microscopy determines the external microstructure and morphology of film samples of 5 × 5 mm^2^. The dried film was placed into liquid nitrogen to remove any moisture content and then ruptured. Then, the ruptured film was snorted with gold using an ion sputter. Finally, a sample kept the FE‐SEM (Jeol JSM‐7001F, Moscow) slot with imaging at 20 kV.

#### 2.4.7 X‐Ray Diffraction (XRD) measurements

The X‐ray diffraction (Rigaku Ultima IV, China) patterns were analyzed by diffractometer using a Cu tube operated at 40 kV and 30 mA. Operating parameter kept at step size 0.02° 2θ with scan time 0.5 S/step with diffraction ranging from 4 to 70 2θ.

#### 2.4.8 Antioxidant activity

The antioxidant activity of whey protein‐based films was analyzed based on the principle of scavenging the stable DPPH. The method described by Salvia‐Trujillo et al., 2015 shows that 0.1g of film sample was deposited in a flask containing 2ml of ethanol and vortex for 2 min and centrifuged for 30 min at 4293 *g*. 0.5 ml of the upper solution was collected and added to 2ml of methanol DPPH solution and 1 ml of ethanol. The mixture was mixed by vortex and keeping at a dark place for 30 min with ambient temperature. The controls were prepared with the same procedure but without film. Using a UV‐spectrophotometer (Shimadzu UV‐2700, Japan), the sample at 517‐wavelength absorbance was measured against the blank sample.

DPPH scavenging activity was calculated by the following equation 3.

$$\text{DPPH scavenging activity}\left(\%\right)\;=*100\frac{\text{Abs control}‐\text{Abs sample}}{\text{Abs control}}*100*100\left(3\right)$$

#### 2.4.9 Release of sunflower oil from the bioactive film

Water and films were used to conduct a release test; the film samples were cut into 2 × 2 cm^2^ and wholly immersed in a container of 10ml of distilled water. After that covering, the samples were kept in darkness at different temperature levels—25°C and 4°C. The absorbance of released sunflower oil was assessed every day for 1 week, according to the standard using UV spectrophotometer of film sample recorded at wavelength 318 nm.

##### Mechanical properties

The Dynamic mechanic analyzer test (Netzsch DMA 242C, Moscow) includes the tensile strength, Youngs modulus, and elongation at break. The film sample of length 110 and width 15 mm. Force up to 0.5 kN; frequency of simultaneous data recording up to 1 kHz for load, elongation, and deformation channels; speed range from 0.05 to 2500 mm/min.

##### Antimicrobial activity tests

According to the report by Pathak et al., 2015, two types of bacteria were used, gram‐positive (*S. Aureus*,) and gram‐negative (*Escherichia coli*) bacteria were used as testing microorganisms. Antibacterial activity of whey protein‐based film samples was evaluated using the zone inhibition test. Many researchers have also adopted this test for the antimicrobial study as it is quick and easy to measure. Different sunflower nanoemulsion concentrations base on whey protein concentration (Code 12.5 = 0.5NE, 25 = 1NE, 50 = 2.5NE, and 100 = 5 NE ) were placed against *E. coli and S. Aureus* for the microbial activity and incubated at 35ºC for 24 h.

### 2.5 Statistical analysis

The data are expressed as mean ± Standard Error of Mean (SEM) for each group. Statistical analysis was done using GraphPad Prism version 8.0 software (GraphPad software, 2019). Value in columns (Table 1 and 2) with standard deviation has the significance of analysis (*p* ≤ .05).

**TABLE 1:** Water vapor permeability, swelling ratio, and moisture absorption

		Control film	10%NE film	25%NE film	50%NE film
Water vapor permeability	Tween80	3.4 ± 0.013 × 10^–10^	2.	2.4 ± 0.011 × 10^–10^	1.9 ± 0.012 × 10^–10^
Moisture absorption (%)		22.5 ± 0.02	17.5 ± 0.012	14.5 ± 0.01	11 ± 0.013
Swelling ratio (%)		7 ± 0.09	5.6 ± 0.088	4 ± 0.01	3 ± 0.097
DPPH	Tween20	–	29	38.7	52.03
	Tween80	–	27	36	50

**TABLE 2:** Thickness and mechanical properties of control and nanoemulsion containing film

Sample	Thickness (mm)	Tensile strength (MPa)	Youngs modulus (MPa)	Elongation at break(%)
Control	0.21 ± 0.02	2.75 ± 0.3	25.22 ± 1.2	10.4 ± 1.2
10 NE	0.22 ± 0.01	3.45 ± 0.2	28.55 ± 1	11.2 ± 0.96
25 NE	0.23 ± 0.02	3. 52 ± 0.3	29.14 ± 0.7	15 ± 0.3
50NE	0.22 ± 0.02	3.50 ± 0.18	28.27 ± 1.24	13.2 ± 1.44

## 3 RESULTS AND DISCUSSIONS

### 3.1 Stability of the formed nanoemulsion

**FIGURE 1:**
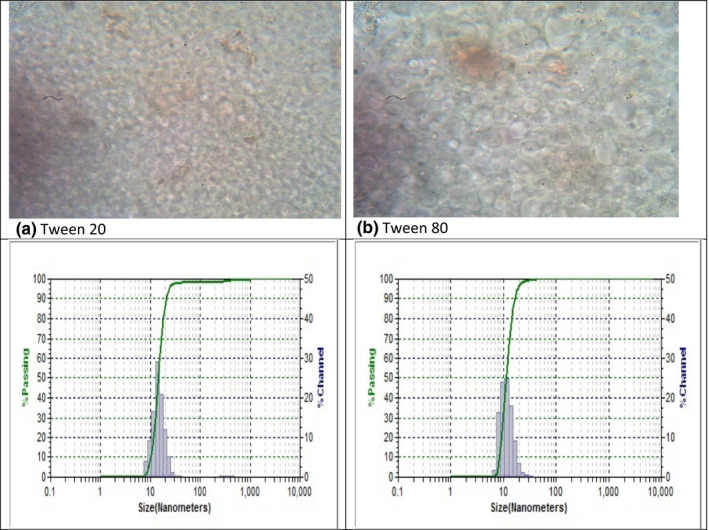
Microscopic image of nanoemulsion with particle size distribution (a) Tween 20 and (b) Tween 80

Before examining the nanoemulsion's effect on the film's success, it is critical to examine its stability. Tween 80 and Tween 20 synthetic sunflower nanoemulsions have droplet sizes of 11.5 and 14.30 nm, respectively. Furthermore, Zeta potential is a technique used to analyze the surface charge density between particles in order to predict coagulation stability. This measurement provides detailed information about various stability phases, such as flocculation and aggregation. The result is a nanoemulsion with droplets with polydispersity indexes of (PDI = 0.104 and 0.12) and a Zeta potential of (−21 mV). Figure 1 shows a perfect encapsulation of sunflower oil in water morphology. The emulsifier layer surrounded the oil particles in a stable form, with no aggregation. These oil droplets are so small that they disperse completely in the nanoemulsion. Based on the findings, the nanoemulsion was incorporated into the film matrix for further investigation.

### 3.2 Films’ characterization

The whey protein‐PVA film blend is prepared with and without sunflower nanoemulsion oil. Such films are typically homogeneous, transduced, and brownish. The films contain a higher concentration of nanoemulsion oil (50% NE) and are more visually flexible than the other films. The color was determined by the dyes used in the industry's production of whey protein isolate. The type of surfactant and the concentration of sunflower used influenced the thickness of the film. When compared to the film formed with Tween 80, the film formed with Tween 20 has a smaller thickness. According to the research, the thickness of the films increases as the alkyl moiety increases. Tween 20 has a low molecular weight, allowing it to form the thinnest layer with its matrix or substrate. Tween 80, on the other hand, has a long molecular weight and one unsaturated double bond, which causes the molecules to lie parallel to the substrate. Because of the high concentration of vegetable oils, such as sunflower, the thickness of the films increased as the concentration of vegetable oils increased. As a result of this study, the thickness of the produced films increased as the concentration of sunflower increased. (see Table 2).

### 3.3 Water vapor permeability for control and nanoemulsion film

**FIGURE 2:**
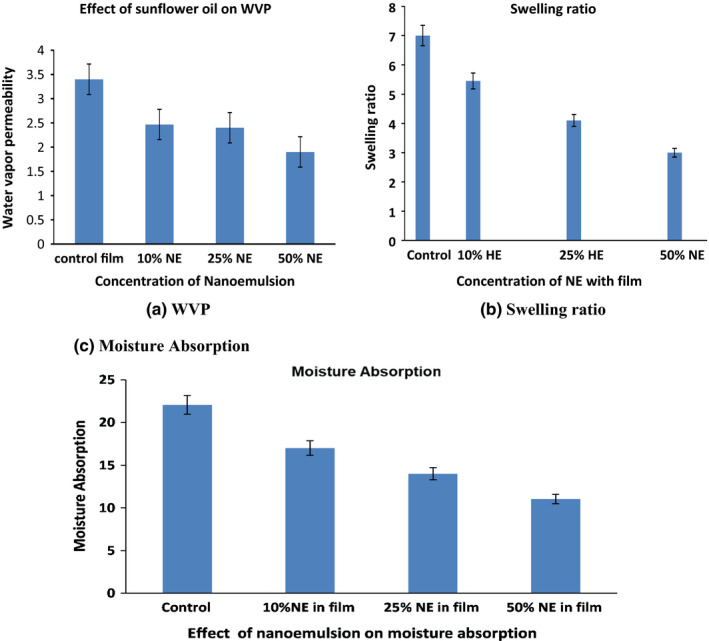
Control and nanoemulsion‐based whey protein active film (a) WVP (b) Moisture absorption, and (c) Swelling ratio

The role of packaging is to prevent the deterioration of the packaged product; the water vapor permeability of the packaging film should be considered. Water vapor permeability is a degree of moisture pass through the active film in terms of weight absorption concerning time. It is an indicator for film, which can absorb the quantity of water vapor at a unit of the area within a given time. One of the significant features to mark suitable food packaging is the water vapor permeability property. The film should be accomplished to hinder the moisture transfer from the atmosphere to the film material. Figure 2a and Table 1 show the result for water vapor permeability of the control film and nanoemulsion containing film samples, and in the ranges from 1.9 × 10^−10^ g/m.h. Pa to 3.4 × 10^−10^ g/m.h. Pa. The results show a decreasing pattern by increasing the concentration of sunflower nanoemulsion in the matrix film formulation. The addition of nanoemulsion improve WVP of film, as they are the significant difference (*p* < .05) in value with same order of magnitude (the Value of WVP for control film is 3.4 × 10^−10^ g/m.h. Pa reduce to 1.9 × 10^−10^ g/m.h. Pa with the addition of nanoemulsion). Increasing the concentration of nanoemulsion in a film means increasing its hydrophobicity. However, a contradictory part, the occurrence of even dispersed and orderly droplets of nanoemulsion in the film structure, causes the moisture particles to pass through in a curved path, reducing the movement rate of moisture particles. In Tween 80 base film shows less water permeability than control film because the alkyl group was observed to be more in control film. As mentioned in section 3.2, the thickness of Tween 80 film is more compared to control film. As a result, as thickness increases, the water vapor permeability also increases.

### 3.4 Swelling ratio

The swelling ratio is a noble quantity for defining the water‐dependent assets of biopolymer material or films. The film matrix swelling may show a significant part in releasing the oil droplet from the film matrix. Consequently, by regulating the swelling ratio, the release rate would remain meticulous. As shown in Figure 2b and Table 1, the control film without nanoemulsion had the maximum swelling(7%), whereas for 10%, NE film having 5.6% and subsequently reduce 3% for 50%NE film (*p* < .05). The reduction in swelling ratio because of reduction in resistance to water absorption of existence of polar chemical groups and the decreased free volumes of polymer molecule relaxation. The possible reason is that the control film is hydrophilic and contains more hydroxyl groups in their structure, mainly glycerol and whey protein. However, because nanoemulsion is hydrophobic, it reduces the mobility of water absorbed onto the whey protein film medium. The use of nanoemulsions to reduce the size of encapsulated oil globules results in a uniform distribution of droplets. As a result, the water or moisture restored in vacant lumens or microvoids in the blend film matrix can be reduced.

### 3.5 Moisture absorption

Moisture/water absorption is the capacity of plastic or a biopolymer to absorb moisture from its environment. Results are presented in Figure 2c and Table 1; there is a reduction in moisture absorption in the film with sunflower oil. The moisture absorption value for control film is 22.5%, which reduces to 17.5% for 10% NE addition to the film and subsequently reduces to 11% for 50% NE film (*p* < .05). The control film contains a combination of whey protein isolate and polyvinyl alcohol, both of which have many hydroxyl groups in their chain. Increases the plasticizing effect of the control film, resulting in a void or pore in the film structure as seen in the SEM image, causing more water to be absorbed on its film. At the same time, an emulsifier with a hydrophilic tail on the exterior surface stabilizes the nanoemulsion‐based film. As a result, there is a chance of forming a hydrogen bond with a free hydroxyl group in the film, resulting in the reduction of a hydroxyl group for reaction with water. Despite the large amount of hydroxyl groups in polyvinyl alcohol, it is assumed that these hydroxyl groups remained connected to the whey protein chains. The SEM analysis results show a strong interaction between whey protein isolate and polyvinyl alcohol, which may be related to the formation of hydrogen bonds.

### 3.6 Morphology observation by FESEM

**FIGURE 3:**
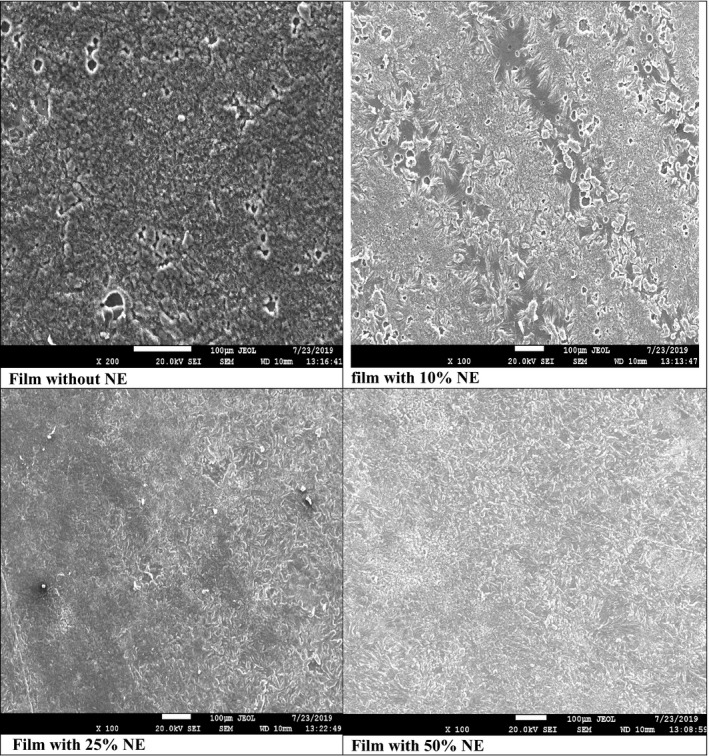
FE‐SEM of control and nanoemulsion‐based whey protein active film

The structural morphology of nanoemulsion films was analyzed by using FESEM. In Figure 3, all images are surface‐sectional images of the films with or without nanoemulsion of sunflower. Film shows roughness and cavities in the cross‐sectional area with or without NE. The control whey protein isolate, polyvinyl alcohol film, had a more rough surface showing a solid interaction between whey protein isolate and polyvinyl alcohol. However, the addition of nanoemulsion reduces the surface irregularity of the formed films. However, the film with 10% NE shows some irregularity in its structure, but increasing the NE content reduces the film cavities or pore and roughness. This reduction in irregularity may be correlated to emulsifiers, especially Tween, which creates hydrophilic interaction with the matrix. Such interaction increases the integrity of the blend of whey protein isolate with polyvinyl alcohol. Mainly, the outcomes of the SEM test observe that the addition of sunflower nanoemulsion has not a negative impact on the density and structural integrity of the film. However, it may contribute to the film's compactness and smoothness, which is already observed in images.

### 3.7 FT‐IR analysis of film containing whey and nanoemulsion

**FIGURE 4:**
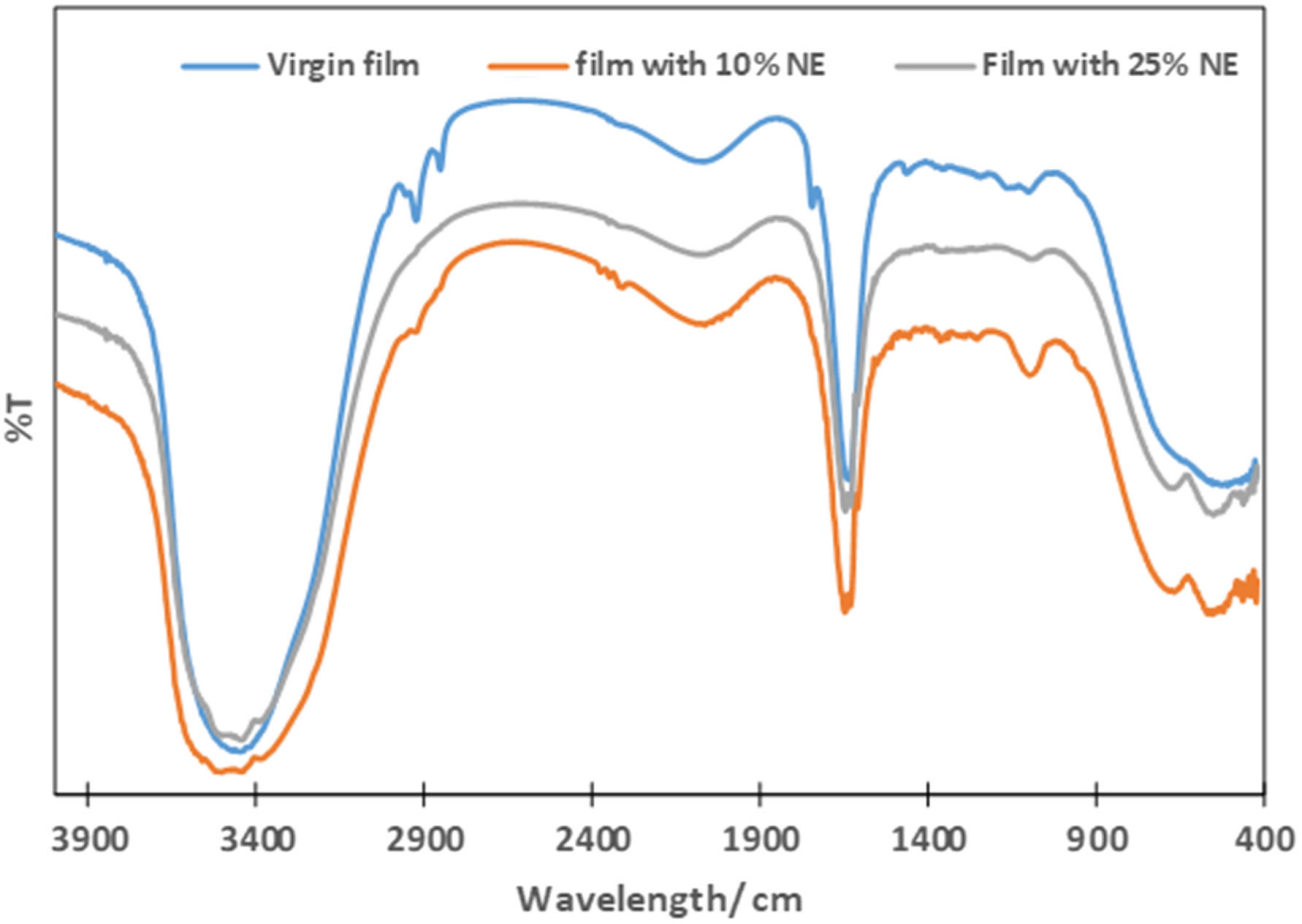
FTIR of Virgin (control) and nanoemulsion film

Many absorption peaks demonstrated FT‐IR results of the control of films based on whey protein and nanoemulsion. As we observe from Figure 4, the absorption peaks ranging from 500 to 750 cm^−1^ are associated with glycerol groups along with the H‐H bond ranging from 900 to 1300 cm^−1^. In addition, there is a peak of bending amide and vibration bond of carboxyl and C‐H at range 1600–2150 cm^−1^, respectively. Later, we observed peaks starting at 2950 cm^−1^ associated with the –H valence bond and O–H and N–H bonds associated at peak 3200–3650 cm^−1^ for all film samples. The same results were obtained in a film prepared from whey protein containing Bioss nanoemulsions. The addition of a sunflower nanoemulsion led to a significant change in the peak of the Virgin Film (control). The addition of a nanoemulsion changed at peak intensity in the range 2900 cm^−1^ of C‐H bonds. When sunflower nanoemulsions are added to the primary film, they decreased and disappeared. Another peak slightly changes (near 3450 cm^−1^) which is associated with the O–H group of protein molecules that disappear due to the inclusion of sunflower nanoemulsions. Thus, the homogeneous distribution of Tween 20 at the oil–water interface causes this peak to disappear in nanoemulsion films of incorporated samples. A decrease in the concentration C–H and O–H bond shows that nanoemulsion mixtures of sunflower are capable of binding to the matrix of biopolymers and maintaining a chemical bond within the biopolymer. The result shows that a sunflower nanoemulsion can remain in the complex of a mixed film based on whey protein due to physical capture and the establishment of chemical bonds.

### 3.8 X‐ray Analysis (XRD)

**FIGURE 5:**
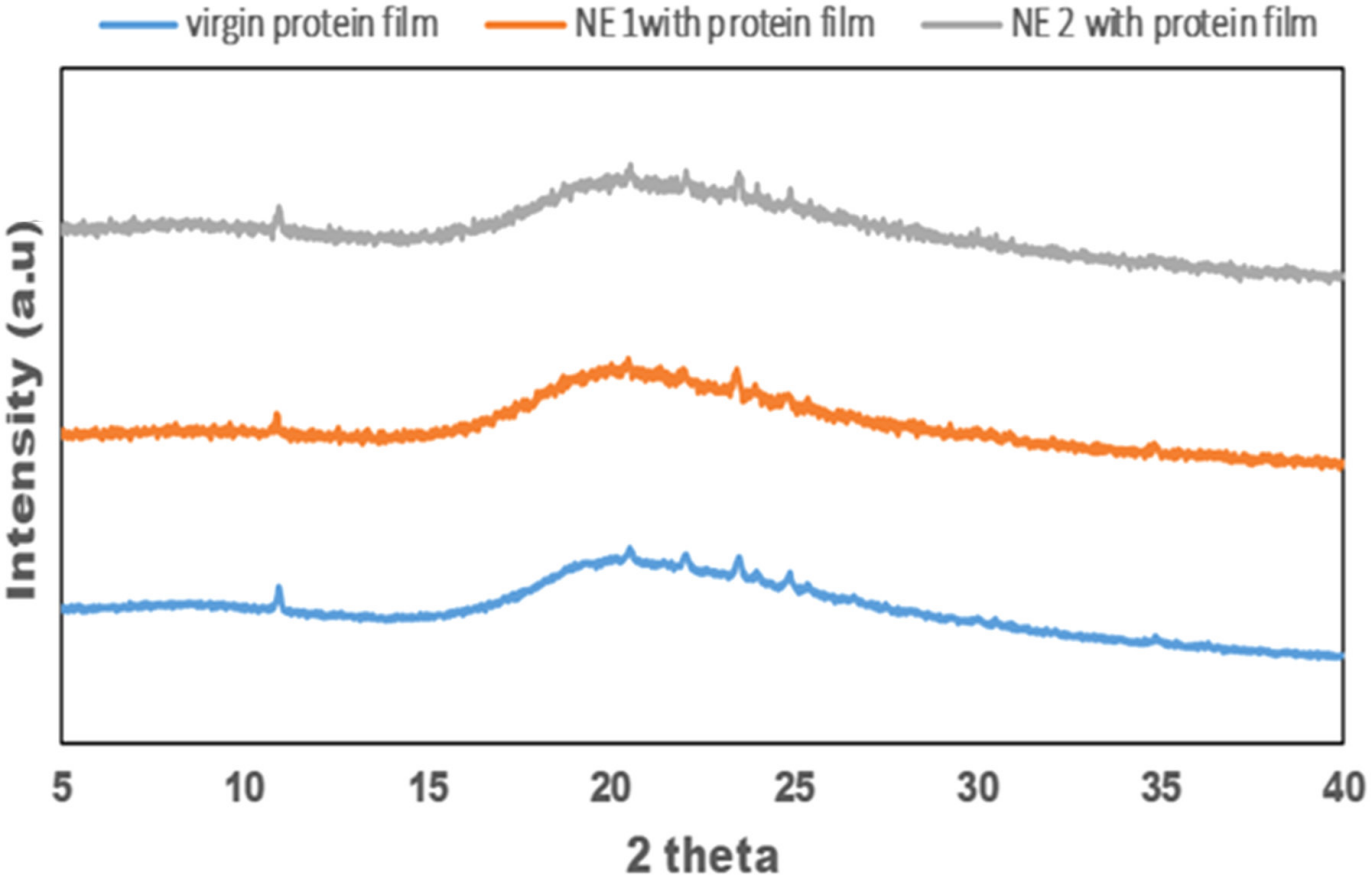
XRD of Virgin (control) and nanoemulsion film

In Figures 5, X‐ray diffraction patterns of a control (virgin) protein‐based film without the introduction of sunflower and films containing 25% and 50% of sunflower nanoemulsions. The control film demonstrates two precise peaks at 2θ at 10° and 20°. The result would designate that whey protein has a moderate crystalline degree. So, it can consider a semicrystalline biomatrix. The intensity of all was not affected by introducing a nanoemulsion of sunflower oil (*p* < .05). This wealth that the addition of nanoemulsion to film matrix does not affect its crystalline nature film structure. The stabilized drops of Tween 20 and Tween 80 can generate more bonds with a film matrix of whey protein.

### 3.9 Release of oil from the active film with nanoemulsion

**FIGURE 6:**
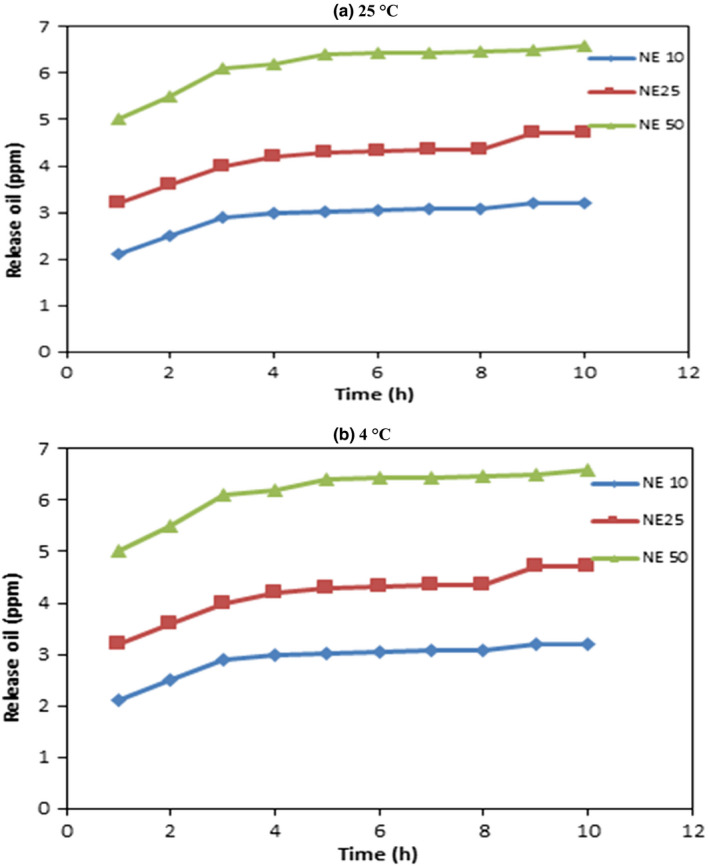
Release of oil from film at different condition (a) 25℃ (b) 4℃

The diffusion process is trailed by the relocation of phenolic compounds from the matrix to a fluid medium. Diffusion occurs when a substance moves through a membrane from a high to low concentration gradient until the migrant compound reaches the equilibrium state depicted in Figure 6. The amount of oil released from the whey protein nanoemulsion‐based blended film varies with concentration. The release of sunflower oil from 50% NE film is 20 ppm compared to 10.5 ppm oil release for 10% NE film at 25℃, whereas reduction of oil release at 4℃ for 50% and 10% NE film is 6.5 and 3 ppm, respectively. The release rate is higher for the maximum concentration of NE. As we have seen in sections 3.4 and 3.5 that strong interaction between whey and glycerol increases the plasticizing effect, increasing water molecules' mobility. Once the nanoemulsion is incorporated into this film, the mobility also increases, and the release of oil would be higher. The release of the oil from films increases by increasing the addition of nanoemulsion and by increasing the temperature. Generally, particles diffusely associate with the kinetic energy of particles, as shown in Figure 6 at 25°C the release of oil is more than 4°C. The main reason is that if the temperature increases, the kinetic energy associated with them also increases. As the kinetic energy engaged in the film tends to increase the mobility chain between whey protein isolate and PVA, such a condition makes the oil particles move faster from the film matrix and possibly diffuses from it. Another possible reason is that an increase in temperature causes the mobility of the chain that carries the oil droplets along with them and finds their way to pass through the film matrix.

#### 3.9.1 DPPH radical‐scavenging activity

**FIGURE 7:**
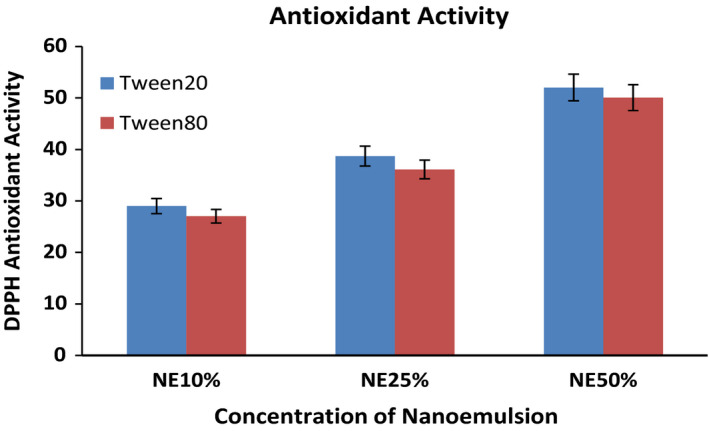
DPPH antioxidant of film containing nanoemulsion at different concentrations

DPPH (2,2‐diphenyl‐1‐picryl‐hydrazyl‐hydrate) is a free radical used as scavenging material in the presence of antioxidants by observing hydrogen molecules. The antioxidant activity of the sample film is analyzed by using a radical scavenging method in terms of DPPH. All the results are shown in Figure 7 and Table 1; the antioxidant activity of 10% and 50% NE film is 27 and 50%, respectively, based on Tween 80 stabilize nanoemulsion, whereas Tween 20 stabilize not much significant difference in their antioxidant activity (*p* < .05). Antioxidant activity of all NE film associated with the content of phenolic compound existing in sunflower oil. The Hydroxyl group presented in the phenolic compound acts as a proton supplier to free radicals. There is a significant increase in antioxidant activity as the concentration of sunflower nanoemulsion increases related to the increase in the concentration of the phenolic compound in the sample films. The films containing nanoemulsions with Tween 20 have more antioxidant activity than those containing Tween 80. The particle size of nanoemulsions can explain this as heavier particles move more slowly and would be a slower diffusion rate. Smaller particles would be diffuse faster because their movement is fast. According to this sunflower nanoemulsion of small average particle size for both NE, the sample shows that droplets move fast and diffuse fast in the solution for antioxidant activity.

#### 3.9.2 Mechanical Properties of different concentrations of NE in protein film

From Table 2, we can see the result of Tensile strength (TE), Young's modulus (Y), and elongation at break (EB) for a prepared film with and without the addition of NE. The thickness of all film samples shows an average of 0.22 mm with a significant difference of *p* < .05. The result shows that films with NE are properly dispersion in it. The film without NE shows fairly good mechanical properties for pure protein as TE, Y, and EB values, 2.75 and 25.22 MPa, and 10.4%, respectively. However, the addition of 10% NE to the films increases the tensile strength value from 2.75 to 3.45, yield increase from 25.22 to 28.55 MPa, with the significant difference (*p* < .05) and elongation at break from 10.5 to 11.2 (there is nonsignificant difference), respectively. Again, further increasing the concentration of NE from 10% to 25%, the value of TE, Y, and EB are all increased to 3.52, 29.14, and 15%, respectively. For value of TE and Y, there is nonsignificant difference, and for EB value there is an increase to 15% (with a significant difference of *p* < .05). The possible reason is that incorporating NE in the film would increase the plasticizing effect of droplets (elasticity) that cause a considerable number of more alkyl groups present in it and its hardness, as shown in the swelling absorption ratio. However, a further increasing maximum concentration of NE (50 NE) shows a slight decrease in the TE, Y, and EB values with a significance of *p* < .05. Because at a specific concentration of NE molecules, the intermolecular bridge between protein molecules reduces the film's strength. In such a situation, this reduction in molecular density of film causes the brittleness of film, which means it loses its flexibility and tensile strength. However, such impact would consider less compared to the result observed for cinnamon essential oil‐based emulsion incorporated in biopolymer material (Bahram et al., 2014; Ramos et al., 2012).

#### 3.9.3 Antimicrobial properties

**FIGURE 8:**
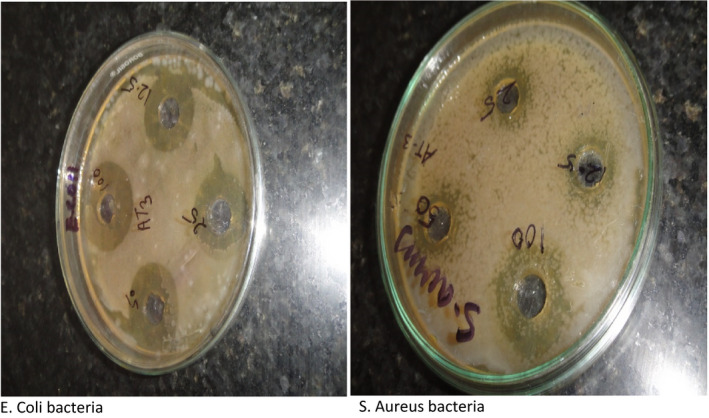
Antimicrobial activity of film containing nanoemulsion at different concentrations

Sunflower oil has been reported to act as an antimicrobial agent. Consequently, it was predictable that the film matrix would have an antimicrobial action. All results show the inhibitory strength of whey protein isolates blended with nanoemulsion. As shown in Figure 8, the incorporation of nanoemulsion to virgin film matrix increases mechanical strength and provides better antimicrobial properties. These trends are increasing with the increasing concentration of nanoemulsion to film matrix. The inhibition zone diameter for different concentrations ranging from 12.5% to 100% NE against *E. coli* 8.2, 9.5,11, and 14 mm and against *S. aureus* 3, 5, 7, and 11, respectively. It is expected that the presence of phenolic complexes in the sunflower oil displays critical antimicrobial activities. The result may be predictable to the release actions of the nanosize droplets of nanoemulsion, which enhances the cellular absorption mechanism and reduces the mass transfer resistance that results in faster penetrating antimicrobial compounds in cell materials. Another point in the figure shows that the antimicrobial activity of the sunflower oil on *S*. *aureus* is considerably less than the already examined microorganisms like *E. coli*. This is due to the difference in bacterial structure *E. coli* membrane which show *E. coli* membrane cell has a less complex structure than *S. aureus* membrane. In buildup, *E. coli* has a lipopolysaccharide and peptidoglycan layer that reduces the moisture or oxygen permeate through the film matrix. This causes the increase in antimicrobial action of *E. coli*.

## 4 CONCLUSION

We conclude that nanoemulsion‐based whey protein active packaging film is formed using FTIR, XRD, and *SEM* analysis. It was observed that films did not change their morphology and crystallinity as they show uniform assembly as that control film. These active films show a reduction in moisture absorption and water vapor permeability compared to control film while improving its mechanical properties and antioxidant properties. The antimicrobial properties show a better result for active film compared to control film.

## CONFLICT OF INTEREST

There are no competing interests.

## ETHICS APPROVAL AND CONSENT TO PARTICIPATE

Not applicable. There is no study on animal or human objective.

## CONSENT FOR PUBLICATION

All authors agreed with this publication.

## Data Availability

The datasets generated for this study are available on request to the corresponding author.
